# Public perception and willingness to accept somatic gene therapy: A Belgian survey study

**DOI:** 10.12688/openreseurope.21462.2

**Published:** 2026-01-29

**Authors:** Phaedra Locquet, Margaux Reckelbus, Eva Van Steijvoort, Pascal Borry, Bram Korbmacher, Sofie Gordts, Lauren Vanceer, Isabelle Huys

**Affiliations:** 1 Department of Pharmaceutical and Pharmacological Sciences, KU Leuven, Leuven, Flanders, 3000, Belgium; 2 Department of Public Health and Primary Care, Centre for Biomedical Ethics and Law, KU Leuven, Leuven, Flanders, 3000, Belgium

**Keywords:** Gene therapy, public acceptance, curative therapies, ethics

## Abstract

**Background:**

Genetic disorders affect millions worldwide, yet fewer than 10% of patients currently receive effective treatment. While gene therapies offer significant promise, their clinical translation is hindered by technical, regulatory, and societal challenges. Low enrolment rates in clinical trials, ethical concerns surrounding inclusion criteria, and uncertainty about preventive applications all contribute to slow progress. Public perception plays a crucial role in shaping trial participation and the integration of gene therapies into healthcare systems. This study examines public attitudes in Belgium to support the responsible development and implementation of gene therapy trials.

**Methodology:**

A cross-sectional online survey using convenience sampling was conducted in Belgium with adults (18+) recruited through local pharmacies. The survey included 12 items assessing self-reported knowledge of gene therapy and willingness to accept gene therapy. To evaluate willingness to accept, hypothetical vignettes were used, which varied by treatment characteristics (e.g., side effects, efficacy, limited evidence), patient age (5, 20, 65 years), and symptomatic status (symptomatic, asymptomatic with uncertain or expected future symptoms). Descriptive statistics summarised all items included in the questionnaire.

**Results:**

The sample included 289 participants, of whom 67% had completed higher education, 64% had children, and 87% had heard of gene therapy before. Overall willingness was high. Attitudes were generally positive, with limited concerns about its experimental features (e.g., unknown side effects (12%), long-term effects (8%),and uncertain effectiveness (8%)). However, key barriers included fears of altered identity (39%), external pressure (38%), and skepticism about its novelty (31%). Uncertainty about symptom development consistently reduced willingness. Patients’ age played a secondary role, with younger individuals generally received higher support for gene therapy than older adults.

**Conclusion:**

Public attitudes toward gene therapy were largely positive, guided by perceived benefits over scientific certainty. Support favored curative over preventive use, with participants balancing autonomy and medical guidance in shared decision-making.

## Introduction

Genetic disorders affect millions of people worldwide, yet effective treatments are available for only a small subset of patients ^1^. Advances in gene editing technologies, such as CRISPR-Cas9, enable precise genome modifications ^2^, making somatic gene therapy (a treatment targeting non-reproductive cells) a promising approach for inherited and acquired diseases. Although the initial introduction of gene therapies during the early 1990s was met with considerable optimism, this enthusiasm was tempered by a series of disappointing clinical trials outcomes. Many early experimental therapies failed to demonstrate clinical benefits or resulted in unexpected toxicities, some of which led to widely publicized patient deaths ^3^. Progress over the past decade in safety, delivery, and gene transfer has enabled regulatory approval of several therapies ^3^.

Despite its therapeutic potential, translating somatic gene therapy into clinical practice still faces several challenges. Because many gene therapies target very small, rare disease populations, and because these patients often experience significant delays in obtaining an accurate diagnosis while facing life-threatening conditions, clinical trial recruitment is inherently limited. Beyond these structural constraints, low participation in clinical trials remains persistent, as many eligible individuals hesitate due to unknown long-term effects, adverse side effects, and the therapy’s experimental status ^4^. At the same time, researchers face complex ethical and methodological challenges in defining inclusion and exclusion criteria. Determining whether to enrol children, older adults, or patients at different disease stages involves ethically sensitive and context-specific considerations. These decisions can have far-reaching consequences, influencing both the success and generalizability of clinical trials, as well as public trust in the research process ^5^.

Patients with progressive genetic conditions stand to benefit significantly from gene therapy designed to halt or slow down disease progression. Administering such therapies at an early stage could not only prevent severe, potentially life-threatening complications, but also serve as a preventive intervention to limit further damage ^6^. Although gene therapy is most often presented as a curative treatment, a framing that remains debated given limited long-term follow-up, its effects are currently considered potentially durable, and attitudes towards its preventive use remain unclear ( https://doi.org/10.1007/s11019-024-10223-w). While there tends to be stronger public support for treating symptomatic patients, especially in severe or life-threatening conditions, there is greater ambivalence regarding treatment of asymptomatic individuals genetically predisposed to disease later in life ^7^.

Over the years, numerous studies have explored public attitudes toward gene therapy, with a particular focus on perspectives concerning germline-editing, human enhancement and disease-specific applications. A systematic review (2020) examining the publics acceptability of gene therapy and gene editing found the highest support for applications addressing serious conditions without alternative treatments. However, this support was often tempered by concerns about the long-term safety, potential unknown side effects, and broader ethical implications of genetic modification ^8^. Studies in countries such as Germany, France, and Italy reveal a similarly complex picture, with participants expressing both optimism and caution, often noting concerns about the novelty of the technology and its potential for misuse ^9^.

These findings suggest that public perception toward gene therapy and related technologies depends on context. Surveys and public engagement initiatives have demonstrated the importance of integrating societal perspectives early in the research and development process. Such initiatives not only improve transparency and foster public trust but also help to identify potential sources of resistance or misunderstanding that could hinder clinical progress. Moreover, they also allow for a more inclusive approach to science and innovation, in which societal values and ethical considerations are integrated into research design and governance structures ^7, 10, 11^. The success of early-phase clinical trials, participant recruitment efforts, and eventual incorporation into healthcare systems heavily depend on how these therapies are perceived and understood by the general public ^12^.

In Belgium, public attitudes toward somatic gene therapy remain relatively underexplored. Given the country’s active involvement in biomedical research and its central position in EU-level regulatory processes, it is essential to investigate how the public understands and evaluates these emerging technologies.

This study aims to address this gap and support clinical trial design, policy, and public communication for socially responsive governance of gene therapy in Belgium and Europe.

## Methods

This study involved a cross-sectional online survey conducted using convenience sampling among individuals living in Flanders.

### Questionnaire development

The questionnaire assessed participants’ willingness to accept gene therapy; attitudes toward somatic gene therapy, focusing on the conditions under which participants would consider its use and the factors influencing its acceptability (data file 1) ^13^. The 12-item questionnaire covered the following subcategories: i. Socio-demographic information, ii. Self-reported knowledge of gene therapy, followed by a brief informational video introducing gene therapy, iii. Willingness to accept gene therapy across various hypothetical vignettes, and iv. Attitudes towards the ethical considerations. The informational video (2m17s, data file 2) ^13^ supported sections three and four of the questionnaire and was based on information from ErfoCentrum, an organisation providing reliable and independent health information ^14^. More specifically, the animated video provided information on the nature and modification of genetic and the origin of genetic disorders. It also explained how gene therapy targets the genetic mutation, the use of (viral) vectors and the therapeutic aim of the treatment.

To evaluate willingness to accept gene therapy, section three of the questionnaire presented a series of hypothetical vignettes that varied by treatment characteristics (e.g., side effects, efficacy, limited evidence), patient age (5, 20, 65 years), and symptomatic status (symptomatic, asymptomatic with uncertain or expected future symptoms). Participants indicated in which scenarios they would be willing to accept gene therapy as a treatment option. Vignette design and key variables were informed by previous research on patient preferences and public attitudes ^11, 15– 17^.

The fourth section of the questionnaire included the assessment of 12 ethical considerations related to gene therapy (e.g., perceived changes in identity, conflicts with moral beliefs and/or nature), as identified by previous research on public attitudes toward gene therapy ^7, 11^. Participants indicated how each factor affected their acceptance using a five-point Likert scale.

A pilot study, involving six Dutch-speaking individuals, evaluated the clarity and comprehensibility of the questionnaire and the informational video. No further adjustments were needed.

The anonymous questionnaire was available in Dutch and administrated either in paper format or through an online platform (Lime Survey Version 6.10.0) ^18^.

### Participant recruitment

Eligibility criteria required participants to be Dutch-speaking adults (18+) that resided in Belgium. A convenience sample was recruited through three public pharmacies in Flanders. Participants were personally invited and could complete the survey on paper, using a tablet, or by scanning a QR-code on a flyer. Data collection took place in December 2024.

### Data analysis

The dataset (data file 3) ^13^ was cleaned prior to data analysis, with responses excluded based on the following criteria: i) questionnaires completed only up to section two, ii) duplicate open-ended responses combined with identical demographic characteristics, and iii) suspected straight lining. To identify potential straight lining, the most frequently selected response option was determined for each group of questions. If this option accounted for more than 85% of responses across all sub-items, the pattern was flagged as a possible indication of straight lining. As the questionnaire included three sections, responses were excluded if more than one-third of these sections met the criteria for suspected straight lining.

Subsequent descriptive statistics were performed using SPSS Statistics software (29.0.2.), to generate frequency distributions.

### Ethics

The study obtained ethical approval from the Ethics Committee Research UZ/KU Leuven (MP032851). The survey was anonymous, and therefore informed consent was not required.

## Results

### A. Socio-demographic characteristics

Of the 375 individuals that accessed the questionnaire, 86 responses were excluded from the dataset during the data cleaning process. The final sample consists of 289 individuals (94 men and 195 women) with a mean age of 47 years (SD= 17.1; 95%CI: 44.8-48.7) ( Table 1). Most respondents had attained higher education (67.1%), had children (63.7%) and identified as Catholic (54.0%). Among those who reported a religious affiliation (n=163), 80% indicated that their religion was either not important (43.6%) or only slightly important (36.8%) in guiding their daily decision-making.

**Table 1.** Socio-demographic characteristics of study participants (n=289).

		N	Valid %
**Sex**	*Male*	94	32,5
	*Female*	195	67,5
**Education**	*No diploma*	8	2,8
	*Secundary education*	81	28,0
	*Higher education*	194	67,1
	*Other (e.g., Ph.D., postgraduate, etc.)*	6	2,1
**Children**	*Yes*	184	63,7
	*No*	105	36,3
**Religion**	*Protestants*	4	1,4
	*Catholic*	156	54,0
	*Muslim*	3	1,0
	*None*	120	41,5
	*Other*	6	2,1
**Importance religion** **(n=163)**	*Not important*	71	43,6
	*Slightly important*	60	36,8
	*Rather important*	20	12,3
	*Very important*	8	4,9
	*Super important*	4	2,5

Before watching the video, most respondents reported having heard of gene therapy (86.5%), but most felt unable to explain it (64.7%). After viewing the video, respondents reported feeling rather (56.1%) or very (21.1%) capable of making an informed decision about receiving gene therapy.

### B. Willingness to accept gene therapy

***1. Treatment characteristics***

Overall, respondents demonstrated a general willingness to accept gene therapy as a treatment across the various vignettes ( Table 2). A substantial proportion reported a willingness to proceed with gene therapy despite uncertainties, including unknown long-term effects (76.1%), uncertain effectiveness (70.9%) and the possibility of unknown side effects (60.6%). Moreover, almost half of the respondents stated that they would not delay initiating gene therapy either until (i.) their condition progresses to a more severe stage (50.5%), or (ii.) until greater clarity regarding these uncertainties became available (47.5%). The majority expressed high trust in their doctor, with 86.2% relying on their recommendation before starting treatment.

**Table 2.** Gene therapy acceptance in relation to treatment characteristics (n=289).

	Strongly disagree	Disagree	Neutral	Agree	Strongly agree
I would agree to gene therapy despite the **unknown** ** long-term** effects.	0,7	6,9	16,3	57,1	19
I would agree to gene therapy despite the risk of possible **unknown side effects** in the future.	0,3	12,1	27	47,8	12,8
I would agree to gene therapy despite current uncertainty about its **effectiveness** and **duration** of effects.	0,0	7,6	21,5	56,4	14,5
I prefer to wait with starting a new therapy until my **illness becomes more severe**.	10,4	40,1	21,1	24,6	3,8
I prefer to wait for **more clarity** about safety, effect, and consequences, even if that means risking worse symptoms later.	7,6	39,8	29,1	21,5	2,1
If I decide to start the treatment, I trust my **doctor’s** ** advice**.	0,3	1,7	11,8	56,1	30,1

*Shading in the tables is used to visualize regions with the greatest participant density (percentage of participants). Results related to the following case: “Suppose you have a genetic disease. The symptoms of your illness strongly affect your daily life. You experience difficulties performing household tasks, have problems with mobility, face limitations in social activities, and you are unable to sustain a full workday. Your condition also worsens with age, increasingly impacting your life. There is now a new gene therapy available which, as the only treatment, can stop the progression of your disease. However, because this therapy is new, scientists still know little about its effectiveness, long-term effects, and possible side effects.”*

***2. Patient profiles***

Table 3 shows that uncertainty about symptom development consistently decreased willingness across all patient profiles, while treatment was more strongly favored for younger adults. More specifically, in pediatric scenarios, opinions on treating asymptomatic children were divided, with 28.1% in expressing support and 38.6% opposing treatment. Support dropped significantly to just 7.8% when symptom development was uncertain. However, when the child was symptomatic and their daily life was affected, support for gene therapy rose sharply to 82.5%.

**Table 3.** Gene therapy acceptance for different patient profiles (n=289).

	Strongly disagree	Disagree	Neutral	Agree	Strongly agree
You are the parent of a **5-year**-old child with a genetic disease, but the child currently has **no symptoms.** Doctors expect future issues.	5,3	33,3	33,3	23,5	4,6
You are the parent of a **5-year**-old child with a genetic disease who currently has **no symptoms**. It is **uncertain** whether symptoms will develop.	22,8	42,5	27,0	7,4	0,4
You are the parent of a **5-year**-old child with a genetic disease who currently **experiences symptoms** that strongly affect daily life.	0,4	2,5	14,7	60,7	21,8
You are **20 years** old and have a genetic disease, but currently experience **no symptoms**. Doctors **expect** future issues.	4,9	30,2	26,0	34,4	4,6
You are **20 years** old and have a genetic disease, but currently experience **no symptoms**. It is **uncertain** whether you will develop any.	16,5	43,5	28,4	10,2	1,4
You are **20 years** old and have a genetic disease that **currently causes symptoms** strongly affecting your daily life. These will worsen over time.	0,0	2,1	7,0	57,9	33,0
You are **65 years** old and have a genetic disease, but currently experience **no symptoms**. It is **uncertain** whether you will develop any	6,7	35,4	27,0	24,6	6,3
You are **65 years** old and have a genetic disease, but currently experience **no symptoms**. It is **uncertain** whether you will develop any.	19,3	42,5	25,6	9,5	3,2
You are **65 years** old and have a genetic disease that currently **causes symptoms** that strongly affect your daily life.	1,1	2,8	11,6	56,1	28,4

*Shading in the tables is used to visualize regions with the greatest participant density (percentage of participants)*.

Similar trends were observed across other age groups. For a 20-year-old adult with expected symptom onset, 39% supported gene therapy, while 35.1% were opposed. Support declined to 11.6% when symptom onset was uncertain, but increased substantially to 90.9% when the individual was symptomatic and their condition was worsening.

Among 65-year-olds, support was lower overall; 30.9% were in favour of treatment in asymptomatic cases with expected symptom onset, and only 12.7% supported it when symptom progression was uncertain. Conversely, 84.5% supported therapy when symptoms were present.

***3. Ethical considerations***

Information-related factors had the strongest positive influence; 92.2% indicated that receiving sufficient information would positively impact their decision, and 89.4% reported that fully understanding the therapy would have a similar effect ( Table 4). Perceptions of novelty and risk were approached with caution. The fact that few individuals had previously received gene therapy was viewed negatively by 30.7%, whereas the use of viruses raised similar mixed reactions (56.9% neutral), reflecting widespread uncertainty regarding technical aspects.

**Table 4.** Gene therapy acceptance in relation to ethical considerations (n=289).

	Very negative	Negative	Neutral	Positive	Very positive
Receiving **sufficient information** about the therapy	0,4	1,1	6,4	60,4	31,8
Fully understanding what gene therapy involves	0,0	0,4	10,2	52,7	36,7
Possible future changes in my identity/personality	7,1	32,2	37,5	15,9	7,4
Passing on **genetic changes** to my children	7,8	18,7	29,3	27,9	16,3
The idea that gene therapy goes **against nature**	6,4	16,6	58,7	12,4	6
**Pressure** from doctors or family members to make a decision	7,8	30,4	39,9	20,5	1,4
The idea that gene therapy goes against my **moral or** ** religious beliefs**	14,1	18	48,4	12,7	6,7
Use of a **virus** in the therapy	2,8	12,4	56,9	21,2	6,7
**Availability** of the therapy in my area	1,8	5,3	41,7	42,4	8,8
The fact that few people have **received** gene therapy before me	3,5	27,2	44,5	18,4	6,4
**Affordability** of the therapy for **society** (actual cost of gene therapy)	2,5	12	50,9	28,3	6,4
**Affordability** of the therapy for **yourself** (personal cost contribution to gene therapy)	1,8	21,2	35	34,3	7,8

*Shading in the tables is used to visualize regions with the greatest participant density (percentage of participants). Results related to the following case: “Suppose you had to decide tomorrow whether to undergo gene therapy. To what extent would the following aspects influence your decision to take or not take the therapy? Here, “negative” means it would lead you to decide not to undergo the therapy, and “positive” means it would lead you to decide to undergo the therapy. “Neutral” means it would have no influence on your choice.”*

External pressure from doctors or family members was mostly perceived negatively, with 38.2% of respondents rating it as a negative influence. Moral or religious objections appeared to have less impact, as nearly half (48.4%) indicated these would not affect their decision. In contrast, views were more divided regarding concerns about potential changes to identity and personality, with 39.3% perceiving this negatively. Reproductive considerations elicited similarly mixed responses, though most participants indicated they would not let it influence their decision (44.2%).

Conversely, economic and practical factors were more positively perceived. The affordability of the treatment to the patient was viewed as a positive factor by 42.1% of participants, and societal affordability by 34.7%. The local availability of the therapy was rated positively by a majority (51.2%).

## Discussion

This survey is the first to explore how the public in Flanders perceives gene therapy. While gene therapy is not new, it remains unfamiliar to most participants. Overall, participants expressed generally positive attitudes despite its experimental aspects. Yet, ethical ambivalence persists around identity, heritability, and external influence, while perceptions of novelty and risk were cautiously neutral. In contrast, clear information and understanding significantly enhanced acceptance.

The experience of symptoms emerged as the most influential factor shaping support. Uncertainty about symptom development consistently reduced willingness across age groups. Whereas, age played a secondary role, with younger individuals receiving higher support than older adults in comparable situations.

### Acceptance shaped by trust and justice

Our study participants were open to the idea of gene therapy, despite acknowledging uncertainties around long-term safety and efficacy. This openness may reflect a kind of pragmatic reasoning: when faced with serious or progressive conditions, individuals often show a greater willingness to accept scientific uncertainty. As Jasanoff *et al.* (2018) argue, public support for gene editing is frequently shaped more by therapeutic intent and perceived benefit than by a demand for complete scientific certainty ^19^. Interestingly, participants were relatively unfazed by the technical novelty of gene therapy. The use of viral vectors or the limited number of prior patients did not significantly deter them, but these factors were not strong motivators either. This cautious ambivalence echoes previous studies, which have found that the public tends to adopt a “wait and see” attitude toward new biomedical technologies ^20^.

When it came to potential risks to identity or future generations, the responses were more mixed. Some participants expressed concern about changes to identity or unintended heritable effects, while others were more neutral. This ambivalence reflects long-standing ethical debates around what it means to “intervene” at the genetic level, especially in ways that could affect one’s sense of self or future offspring ^19^.

Finally, while identity and heritability raised theoretical concerns, it was more practical issues like affordability and availability that stood out as key factors influencing acceptance. This speaks to the importance of justice in how people think about access to healthcare innovations. Practical issues like affordability and availability were more influential in shaping acceptance, emphasizing justice in healthcare access ^21^.

### The importance of autonomy and shared-decision making in gene therapy

Our results point to a common gap between recognition and understanding; people are familiar with the term but lack a clear sense of how gene therapy works. This finding echoes previous research showing that public knowledge of gene therapy is often quite shallow, shaped more by media exposure than by meaningful comprehension ^22^. The educational video appeared to help bridge that gap. After watching it, many participants reported to be able to make an informed decision about whether to take gene therapy as a treatment or not. This suggests that even short, well-designed explanations can improve public grasp of complex scientific topics. As Scheufele *et al.* (2019) emphasize, effective public engagement strategies that clearly communicate complex scientific concepts (such as CRISPR or viral vectors) are essential for fostering understanding and building public trust in emerging biotechnologies ^23^.

Autonomy was another recurring theme. External pressure (whether from family members or doctors) was generally perceived negatively. This reinforces the idea that, for most people, decisions about gene therapy should be left to the individual. In contrast, moral or religious objections had relatively little impact, with nearly half of respondents taking a neutral stance. This may reflect a broader shift towards secular, individualistic approaches to ethics (at least in the population sampled here) ^7^. However, the role of medical authority came through very clearly, with almost all participants saying that they would rely on their doctor’s recommendation. Even when dealing with unfamiliar or experimental treatments, people still place a great deal of trust in healthcare professionals, which raises questions about how informed consent processes are actually experienced in practice. This highlights the importance of shared decision-making, where patients and their clinicians collaboratively consider their treatment options ^24, 25^. This ensures that patients are well informed about the options available, respects their autonomy and supports ethically sound care. 

### Gene therapy as a preventive treatment: higher promise, lower public acceptance

In line with the latest research on public attitudes toward gene therapies, our findings indicate a generally positive acceptance ^9, 26– 29^. Specially, acceptance rates are higher in hypothetical vignettes where patients are already symptomatic or have a high likelihood of developing symptoms. Conversely, willingness to accept gene therapy decreases in scenarios when the onset of symptoms remains uncertain. This pattern may reflect the same underlying trend observed in earlier research, where gene therapies receive greater public support when intended for life-threatening conditions, rather than less severe or non-life-threatening cases ^7, 27^. In both cases, the perceived severity or immediacy of the condition appears to play a key role in shaping attitudes toward treatment. These patterns suggest that gene therapy is predominantly perceived as a curative rather than a preventive treatment. Present and visible suffering triggers stronger moral intuitions than probabilistic reasoning about future illness, suggesting a public discomfort with preventive interventions in the absence of suffering. This aligns with earlier ethical analyses that highlight the centrality of current suffering in public moral reasoning ^30^.

Current literature comparing preferences between prevention and cure reveals a wide range of attitudes, including no clear preference between both ^31^. Prevention was perceived as more acceptable than cure when aimed for younger individuals, which aligns with our findings showing greater public support for preventative gene therapy treatments in vignettes featuring younger patients ^31^. This is also reflected by Persad *et al.* (2009), who describe a utilitarian approach to resource allocation, prioritizing individuals who are expected to gain more life-years or future potential from medical interventions ^32^.

Furthermore, existing literature on preventive medicine highlights controversial attitudes, with general practitioners’ recommendations emerging as a strong motivator. At the same time, there is notable scepticism towards pharmaceutical companies and academic institutions ^33^. Furthermore, some studies caution against treating asymptomatic carriers due to the potential for adverse side effects and the risk of incurring unnecessary healthcare costs ^34^. Moreover, Meertens *et al.* (2013) found that treatment interventions tend to receive a greater monetary appreciation than preventive measures ^35^, reinforcing the broader trend of higher public support for curative approaches. While gene therapies are not traditionally classified as preventive measures, they may serve a preventive role for individuals diagnosed with a genetic mutation that puts them at risk of developing certain conditions ^36^. In such cases, early intervention with gene therapy could potentially delay or prevent disease onset. These findings suggest that for genetic conditions with variable penetrance, accurate risk assessment and prognosis are essential to justify the consideration of gene therapy as a viable option in the early stages of disease. Furthermore, patients’ needs and preferences must be considered to ensure gene therapies are developed for those willing to receive them. As Doevendans *et al.* (2022) caution, there is a risk of developing therapies for mildly affected patients who may not even seek them, while leaving severely affected patients without options ^37^.

### Limitations

The survey was distributed exclusively in Flanders and in Dutch, excluding French- and German-speaking communities. Recruitment through pharmacies and the use of convenience sampling may have introduced selection bias, as participants visiting pharmacies could have higher health awareness or greater receptivity to medical interventions. Additionally, the sample overrepresented Catholic women and predominantly consisted of highly educated individuals, which may have influenced responses related to information and trust in science. Furthermore, the limited sample size reduces generalizability, and reliance on self-reported data may introduce social desirability bias. Additionally, the informational video was necessarily brief due to the recruitment strategy, focusing primarily on the genetic nature of gene therapies. As a result, detailed information on complex delivery procedures and gene therapy mechanisms was not included, which may have influenced participants’ perceptions.

## Conclusion

While gene therapy remains unfamiliar to many in Belgium, public attitudes were generally positive, driven more by perceived benefits than by scientific certainty. Most participants believed treatment decision should be autonomously; however they would still heavily rely on their doctor’s guidance, highlighting the importance of shared decision-making. Many perceived gene therapy as a curative treatment, with visible suffering prompting stronger support than preventive use in the absence of symptoms. 

## Disclosures

During the preparation of this work the author(s) used GTP-4o mini in order to improve the readability and language. After using this tool/service, the author(s) reviewed and edited the content as needed and take(s) full responsibility for the content of the publication.

## Data Availability

KU Leuven RDR (Dataverse): Replication Data for: Public Perception and Willingness to Accept Somatic Gene Therapy: A Belgian Survey Study. https://doi.org/10.48804/J67GIM. The project contains the following underlying data ^38^: Dateset_Excel.xlsx KU Leuven RDR (Dataverse): Replication Data for: Public Perception and Willingness to Accept Somatic Gene Therapy: A Belgian Survey Study. https://doi.org/10.48804/J67GIM ^38^. The project contains the following extended data: Questionnaire.docx Video_genetherapy.mp4 Readme.txt Data are available under the terms of the Creative Commons Attribution 4.0 International license (CC-BY 4.0)
