# Supplementary material for: Public perceptions and willingness to accept somatic gene therapy: A Belgian survey study
Source: Open Res Eur. 2026 Apr 28;5:319. Originally published 2025 Oct 16. [Version 4] doi: 10.12688/openreseurope.21462.4 (PMC12905533; doi:10.12688/openreseurope.21462.4)
Supplement: Peer review history

# Reviewer report: Reviewer response for version 4

**DOI:** 10.21956/openreseurope.25624.r73456

**Published:** 2026-05-15

**Authors:** Tao Huang

**Affiliations:** 1 Chinese Academy of Sciences Shanghai Institute of Nutrition and Health, Shanghai, Shanghai, China

The authors enrolled 289 participants, of whom 67% had completed higher education, 64% had children, and 87% had heard of gene therapy before. They found that overall willingness was high. Attitudes were generally positive, with limited concerns about its experimental features. However, key barriers included fears of altered identity (39%), external pressure (38%), and skepticism about its novelty (31%). This is an important work to know public perceptions and willingness to accept somatic gene therapy. There are several problems:

1. The sample size was too small. More participants from diverse backgrounds and countries should be included.
2. The authors should specify the somatic gene therapy, since different somatic gene therapies have different risks.
3. On https://rdr.kuleuven.be/dataset.xhtml?persistentId=doi:10.48804/J67GIM and https://rdr.kuleuven.be/dataset.xhtml?persistentId=doi:10.48804/J67GIM, Dataset_Excel.xlsx was locked. The authors should remove the identity and make the data publicly available.

Is the study design appropriate and does the work have academic merit?

Yes

Is the work clearly and accurately presented and does it cite the current literature?

Yes

If applicable, is the statistical analysis and its interpretation appropriate?

Yes

Are all the source data underlying the results available to ensure full reproducibility?

No

Are the conclusions drawn adequately supported by the results?

Partly

Are sufficient details of methods and analysis provided to allow replication by others?

Partly

Reviewer Expertise:

Bioinformatics

I confirm that I have read this submission and believe that I have an appropriate level of expertise to confirm that it is of an acceptable scientific standard, however I have significant reservations, as outlined above.

# Reviewer report: Reviewer response for version 4

**DOI:** 10.21956/openreseurope.25624.r73278

**Published:** 2026-05-07

**Authors:** Eva-Maria Merz

**Affiliations:** 1 Vrije Universiteit Amsterdam, Sanquin, The Netherlands

I think the authors have done a good job and addressed all open points adequately. I have nor further comments.

Is the study design appropriate and does the work have academic merit?

Yes

Is the work clearly and accurately presented and does it cite the current literature?

Partly

If applicable, is the statistical analysis and its interpretation appropriate?

Yes

Are all the source data underlying the results available to ensure full reproducibility?

Partly

Are the conclusions drawn adequately supported by the results?

Yes

Are sufficient details of methods and analysis provided to allow replication by others?

Yes

Reviewer Expertise:

NA

I confirm that I have read this submission and believe that I have an appropriate level of expertise to confirm that it is of an acceptable scientific standard.

# Reviewer report: Reviewer response for version 4

**DOI:** 10.21956/openreseurope.25624.r73277

**Published:** 2026-04-30

**Authors:** Sharon Phares

**Affiliations:** 1 Tufts Medical Center, Boston, Massachusetts, USA

Thank you for addressing the comments on the initial version

Is the study design appropriate and does the work have academic merit?

Yes

Is the work clearly and accurately presented and does it cite the current literature?

Partly

If applicable, is the statistical analysis and its interpretation appropriate?

Yes

Are all the source data underlying the results available to ensure full reproducibility?

No

Are the conclusions drawn adequately supported by the results?

Yes

Are sufficient details of methods and analysis provided to allow replication by others?

Yes

Reviewer Expertise:

healthcare system design, cell & gene therapies, primary care, health services research

I confirm that I have read this submission and believe that I have an appropriate level of expertise to confirm that it is of an acceptable scientific standard.

# Reviewer report: Reviewer response for version 3

**DOI:** 10.21956/openreseurope.25342.r71259

**Published:** 2026-03-25

**Authors:** Eva-Maria Merz, Louisanne van Hooff

**Affiliations:** 1 Vrije Universiteit Amsterdam, Sanquin, The Netherlands; 2 Vrije Universiteit Amsterdam, Amsterdam, The Netherlands

- In the abstract, under Results, a space is missing between ‘long-term effects (8%),’ and ‘and uncertain’.
- In the introduction on page 4, the second paragraph, I want to suggest to the authors to refer to Krishanmurti et al. (2026) ‘Informed shared decision-making or rigid eligibility and exclusion criteria for gene therapy for sickle cell disease?’ as a nice complementary example of the challenges that go with inclusion and exclusion criteria and eligibility considerations.
- In the methods section on page 5, in the second to last sentence of the first paragraph, a word is probably missing after ‘modification of genetic’.
- On page 5, the authors explain that the fourth section of the survey was based on previous research on ethical considerations that influenced acceptability of GT. Currently, I do not understand the choice for the question ’. I do see that it is based on previous literature by Robillard et al. and Wang et al., but what do the authors mean with a change in a person’s identity/personality? This question can be interpreted in so many different ways by a survey participant, that I’m wondering if this question measures what the authors want to know.
- In the methods section on page 5 under Participant recruitment, the authors explain that participants were recruited through three local pharmacies, and in the limitations section, attention has been addressed to the bias this might have introduced due to a possible higher health awareness and greater receptivity to medical interventions. However, it is still unclear why the authors opted for recruitment through local pharmacies in the first place. What was the rationale behind this?
- In the rebuttal letter to the second reviewer, the authors shared their rationale behind choosing the ages 5, 20 and 65. It would benefit the paper to add the rationale behind these ages in the main text as well.
- In table 3 on page 7, the word choice in the scaling from ‘strongly disagree’ to ‘strongly agree’ does not make sense in combination with the statements in column 1. The statements cannot be answered with a ‘(dis)agreement’. It seems a question prompted like ‘would you agree or disagree with a gene therapy in the following cases?’ or something along those lines was asked on the survey, but the reader does not know that right now. Providing extra information on the prompts or wording of these questions or the scaling is necessary. Additionally, the third to last and second to last statements in the first column, currently are exactly the same. I think the wording in the third to last statement was also intended to be ‘Doctors expect future issues’ instead of ‘It is uncertain whether symptoms will develop.’ This needs to be corrected.
- On page 9, the authors start the second full paragraph with ‘Autonomy was another recurring theme.’ No themes are mentioned earlier in the piece, and the methods section does not mention a thematic analysis or anything similar. What do the authors mean with this?
- From the rebuttal letter to the second reviewer, I understand that the authors decided to not include side effects and symptoms, as the goal of the study was to look at perceptions of associated uncertainty and to limit survey fatigue. However, to measure acceptance and perceptions of uncertainty, it would have been very interesting to include information in the video, or a question in the survey on the conditioning that takes place in advance of a GT, on the isolation period, and on the risk of possible future (in)fertility. Moreover, these also fall under ethical considerations, as they carry moral and social consequences of which the importance might shift with a (hypothetical) patient’s age. It might be necessary to better argue why these topics were not included in the video/survey, or add it to the limitations section of the discussion on page 10, in more detail.
- On page 10, the authors mentioned using GTP-4o mini, but I think they meant GPT-4o mini.
- In the questionnaire that is shared on KU Leuven RDR (Dataverse) on page 3 and page 5, there are questions that were not translated from Dutch to English.

Is the study design appropriate and does the work have academic merit?

Yes

Is the work clearly and accurately presented and does it cite the current literature?

Partly

If applicable, is the statistical analysis and its interpretation appropriate?

Yes

Are all the source data underlying the results available to ensure full reproducibility?

Partly

Are the conclusions drawn adequately supported by the results?

Yes

Are sufficient details of methods and analysis provided to allow replication by others?

Yes

Reviewer Expertise:

NA

We confirm that we have read this submission and believe that we have an appropriate level of expertise to confirm that it is of an acceptable scientific standard, however we have significant reservations, as outlined above.

# Response

**Published:** 2026-04-10

**Authors:** Phaedra Locquet

**Affiliations:** KU Leuven Department of Public Health and Primary Care, Leuven, Flanders, Belgium

We thank the reviewer for their careful and thorough review. We have considered all feedback and addressed each point below, incorporating the suggested revisions to improve the manuscript.  

 In the abstract, under Results, a space is missing between ‘long-term effects (8%),’ and ‘and uncertain’.  

**Thank you, we corrected that typo.**

 In the introduction on page 4, the second paragraph, I want to suggest to the authors to refer to Krishanmurti et al. (2026) ‘Informed shared decision-making or rigid eligibility and exclusion criteria for gene therapy for sickle cell disease?’ as a nice complementary example of the challenges that go with inclusion and exclusion criteria and eligibility considerations.  

**Thank you for this helpful suggestion. We agree that this recent paper provides a relevant and concrete illustration of the ethical and methodological tensions surrounding inclusion, exclusion, and eligibility criteria in gene therapy trials. We have now incorporated a reference to Krishanmurti et al. (2026) in the second paragraph of the Introduction (p.4) to complement our introduction with this example from sickle cell disease.**

 In the methods section on page 5, in the second to last sentence of the first paragraph, a word is probably missing after ‘modification of genetic’.  

**Thank you for pointing this out. We have revised the sentence for clarity.**

 On page 5, the authors explain that the fourth section of the survey was based on previous research on ethical considerations that influenced acceptability of GT. Currently, I do not understand the choice for the question ’. I do see that it is based on previous literature by Robillard et al. and Wang et al., but what do the authors mean with a change in a person’s identity/personality? This question can be interpreted in so many different ways by a survey participant, that I’m wondering if this question measures what the authors want to know.  

**Thank you for this comment. We would like to clarify that the question on changes to a person’s identity or personality was intended to capture participants’ perceptions or beliefs about the potential impact of gene therapy, not to suggest that gene therapy can actually alter personality. This approach follows prior studies by Robillard et al. and Wang et al., which similarly explored public perceptions of ethical implications related to personal characteristics in genetic interventions. We acknowledge that participants may interpret this item in slightly different ways, reflecting the complexity of assessing attitudes toward hypothetical scenarios. We have added it to the limitations section:** *“* *The survey item addressing potential changes to a person’s identity or personality may have been interpreted differently by participants, which could affect the precision with which this question captured perceptions of ethical concerns related to gene therapy.”*  

 In the methods section on page 5 under Participant recruitment, the authors explain that participants were recruited through three local pharmacies, and in the limitations section, attention has been addressed to the bias this might have introduced due to a possible higher health awareness and greater receptivity to medical interventions. However, it is still unclear why the authors opted for recruitment through local pharmacies in the first place. What was the rationale behind this?   Thank you for this observation. Participants were recruited through local pharmacies as a pragmatic and accessible setting within the Belgian healthcare system, where community pharmacies serve a broad and diverse segment of the population, allowing efficient recruitment within a limited timeframe.   In the rebuttal letter to the second reviewer, the authors shared their rationale behind choosing the ages 5, 20 and 65. It would benefit the paper to add the rationale behind these ages in the main text as well.  

**Thank you for this helpful suggestion. We have now added the rationale for selecting the ages 5, 20, and 65 years in the Methods section. It reads as follows:** *“* *The chosen age groups were based on the case design of Ormondroyd et al. (2024), who used ages 5, 20, and 50 years, with 50 being replaced by 65 years to better capture differences in life stage, responsibilities, and social context.”*    

 In table 3 on page 7, the word choice in the scaling from ‘strongly disagree’ to ‘strongly agree’ does not make sense in combination with the statements in column 1. The statements cannot be answered with a ‘(dis)agreement’. It seems a question prompted like ‘would you agree or disagree with a gene therapy in the following cases?’ or something along those lines was asked on the survey, but the reader does not know that right now. Providing extra information on the prompts or wording of these questions or the scaling is necessary. Additionally, the third to last and second to last statements in the first column, currently are exactly the same. I think the wording in the third to last statement was also intended to be ‘Doctors expect future issues’ instead of ‘It is uncertain whether symptoms will develop.’ This needs to be corrected.

**We have added the questions indicating how the agree/disagree scale should be interpreted. It reads now as follows:** *Shading in the tables is used to visualize regions with the greatest participant density (percentage of participants)*. *Results related to the following question: “In the following statements, indicate the extent to which you agree with the consideration to undergo gene therapy, taking into account the age and stage of the disease.”.* **Additionally, we thank the reviewer for catching this error in the table, which has since been corrected. The statements have also been revised in line with your suggestions.**  

 On page 9, the authors start the second full paragraph with ‘Autonomy was another recurring theme.’ No themes are mentioned earlier in the piece, and the methods section does not mention a thematic analysis or anything similar. What do the authors mean with this?  

**Thank you for pointing this out. We have revised the paragraph for clarity and now describe the findings directly in terms of participants’ responses rather than referring to “themes.” The revised sentence reads:** “ *Participants’ responses highlighted the importance of autonomy in decision-making*.” This avoids any confusion about thematic analysis while accurately reflecting the data.  

 From the rebuttal letter to the second reviewer, I understand that the authors decided to not include side effects and symptoms, as the goal of the study was to look at perceptions of associated uncertainty and to limit survey fatigue. However, to measure acceptance and perceptions of uncertainty, it would have been very interesting to include information in the video, or a question in the survey on the conditioning that takes place in advance of a GT, on the isolation period, and on the risk of possible future (in)fertility. Moreover, these also fall under ethical considerations, as they carry moral and social consequences of which the importance might shift with a (hypothetical) patient’s age. It might be necessary to better argue why these topics were not included in the video/survey, or add it to the limitations section of the discussion on page 10, in more detail.  

**Thank you for this suggestion. We have added a note to the limitations section acknowledging that the video and survey did not include information on pre-treatment conditioning, isolation periods, or potential impacts on future fertility. We briefly explain that these topics were deliberately excluded to limit survey fatigue and maintain focus on general perceptions of uncertainty associated with gene therapy, while noting that future research could explore these factors in more detail.** *“The informational video was necessarily brief due to the recruitment strategy, focusing primarily on the genetic nature of gene therapies; as a result, detailed information on complex delivery procedures, pre-treatment conditioning, isolation periods, or potential future fertility consequences was not included. These factors may influence participants’ perceptions of uncertainty and acceptance and carry ethical and social implications that could vary with the patient’s age. We deliberately excluded them to limit survey fatigue and maintain focus on general perceptions of uncertainty associated with gene therapy, but future research could explore these additional aspects to better understand their impact on decision-making and ethical considerations.”*  

 On page 10, the authors mentioned using GTP-4o mini, but I think they meant GPT-4o mini.   Thank you, we corrected that typo.   In the questionnaire that is shared on KU Leuven RDR (Dataverse) on page 3 and page 5, there are questions that were not translated from Dutch to English.  

**Thank you, we have made the necessary translations.**

# Reviewer report: Reviewer response for version 2

**DOI:** 10.21956/openreseurope.24792.r68978

**Published:** 2026-02-19

**Authors:** Karine Dube

**Affiliations:** 1 University of Pennsylvania, Pennsylvaniaia, Pennsylvania, USA

Overall Assessment:

 This is a timely cross-sectional study on public perceptions and willingness to accept somatic gene therapy in Belgium. The study is well constructed, and the paper is clearly written. The tables, including heat maps of the results, are well designed. Only minor comments are noted. This reviewer recommends indexing after minor revisions.

 Major:

- Did the authors consider including bivariate and multivariate analyses as well?
- There are several mentions of gene therapy as a treatment, but not as a cure. Should this distinction be clarified or reconciled?

Minor:

- Title: Should “perceptions” be plural?
- Abstract, Results: Ensure extra spacing between “),” and “and.”
- Abstract, Results, last sentence: Please check syntax and completeness: “Patients’ age played a secondary role, with younger individuals generally received higher support for gene therapy than older adults.” Should “received” be “receiving”?
- Methods, Participant Recruitment: “participants that” should be “participants who.”
- Results, A Socio-Demographic Characteristics: “individuals that” should be “individuals who.”
- Results, second paragraph: Consider replacing “felt” with “were” [unable to explain it]. It is difficult to determine what survey respondents *feel*.
- Results, 2. Patient Profiles: The word “in” between “28.1%” and “expressing” is unnecessary.
- Discussion, first sentence: Consider adding “To our knowledge, this survey is the first…” [acknowledging that there have been similar surveys in other settings].
- This reviewer recommends a careful final review of grammar, syntax, and punctuation.

Is the study design appropriate and does the work have academic merit?

Yes

Is the work clearly and accurately presented and does it cite the current literature?

Yes

If applicable, is the statistical analysis and its interpretation appropriate?

Yes

Are all the source data underlying the results available to ensure full reproducibility?

Yes

Are the conclusions drawn adequately supported by the results?

Yes

Are sufficient details of methods and analysis provided to allow replication by others?

Yes

Reviewer Expertise:

Socio-behavioral sciences

I confirm that I have read this submission and believe that I have an appropriate level of expertise to confirm that it is of an acceptable scientific standard.

# Response

**Published:** 2026-03-10

**Authors:** Phaedra Locquet

**Affiliations:** KU Leuven Department of Public Health and Primary Care, Leuven, Flanders, Belgium

We thank the reviewer for the careful evaluation of our manuscript. We have addressed each comment in detail, and our responses are presented below.

 1. We did consider including bivariate and multivariate analyses; however, given the limited sample size, we decided not to perform these analyses to avoid Type-1 mistakes.

 2. We adapted a part in the introduction, in which we clearly mention the distinction between treatment & cure, as illustrated below. We decided to not present gene therapy as a cure to the participants because at this moment it remains debatable if one could consider GT as a cure: **“Although gene therapy is most often presented as a cure, this framing remains debated given limited long-term follow-up data. Nevertheless, its effects are currently considered potentially durable.

 3. We thank the reviewer for the suggestion to improve the reading of our manuscript. We have carefully considered all remarks and have incorporated them into the revised version.

# Reviewer report: Reviewer response for version 2

**DOI:** 10.21956/openreseurope.24792.r68898

**Published:** 2026-02-13

**Authors:** Marilyn Baffoe-Bonnie

**Affiliations:** 1 University of Pennsylvania, Pennsylvania, Pennsylvania, USA

Thank you for taking the time to carefully address these comments. The manusript can now be indexed.

Is the study design appropriate and does the work have academic merit?

Yes

Is the work clearly and accurately presented and does it cite the current literature?

Partly

If applicable, is the statistical analysis and its interpretation appropriate?

Yes

Are all the source data underlying the results available to ensure full reproducibility?

No

Are the conclusions drawn adequately supported by the results?

Yes

Are sufficient details of methods and analysis provided to allow replication by others?

Partly

Reviewer Expertise:

I am an ELSI (Ethical, Legal, and Social Implications of Genetics and Genomics) researcher, with a focus on the lived experiences of individuals undergoing gene therapy and the integration of this therapy into clinical practice.

I confirm that I have read this submission and believe that I have an appropriate level of expertise to confirm that it is of an acceptable scientific standard.

# Reviewer report: Reviewer response for version 2

**DOI:** 10.21956/openreseurope.24792.r68899

**Published:** 2026-01-30

**Authors:** Sharon Phares

**Affiliations:** 1 Tufts Medical Center, Boston, Massachusetts, USA

Thank you for making the suggested changes. The article is interesting and will add to the field.

Is the study design appropriate and does the work have academic merit?

Yes

Is the work clearly and accurately presented and does it cite the current literature?

Partly

If applicable, is the statistical analysis and its interpretation appropriate?

Yes

Are all the source data underlying the results available to ensure full reproducibility?

No

Are the conclusions drawn adequately supported by the results?

Yes

Are sufficient details of methods and analysis provided to allow replication by others?

Yes

Reviewer Expertise:

I have done research and published extensively in the area of gene therapy.

I confirm that I have read this submission and believe that I have an appropriate level of expertise to confirm that it is of an acceptable scientific standard.

# Reviewer report: Reviewer response for version 1

**DOI:** 10.21956/openreseurope.23216.r63845

**Published:** 2025-12-18

**Authors:** Marilyn Baffoe-Bonnie

**Affiliations:** 1 University of Pennsylvania, Pennsylvania, Pennsylvania, USA

Overall Comments

 This was a well-conducted study on an increasingly important topic as gene therapy enters health care spaces. A few minor changes are warranted to clarify the study methods and situate it alongside scholarship on how gene therapy is described.

Introduction

 While the authors accurately note that gene therapy is often described as curative, they may consider briefly acknowledging the ongoing debate in the literature about the appropriateness of this framing.

Methods

 A bit more detail would be helpful to understand whether individuals were presented with information about gene therapy in general or regarding specific conditions. The delivery process for gene therapy varies widely, especially across different conditions, with some requiring significantly more complex procedures (e.g., chemotherapy, extended hospitalizations) compared to others (e.g., those performed in outpatient settings). Additionally, the mechanisms for gene therapy (e.g., gene addition, gene correction, gene silencing) vary widely, which may also change perceptions.

 It was unclear what symptoms, long-term effects, and side effects were presented. Again, these vary across conditions and may impact perceptions of willingness to accept gene therapy.

 Why were 5, 20, and 65 chosen as the ages?

Limitations

 Since recruitment occurred in pharmacies, participants may not fully represent the broader public. Individuals visiting pharmacies may have higher health awareness or greater receptivity to medical interventions, which could potentially influence their responses.

Is the study design appropriate and does the work have academic merit?

Yes

Is the work clearly and accurately presented and does it cite the current literature?

Partly

If applicable, is the statistical analysis and its interpretation appropriate?

Yes

Are all the source data underlying the results available to ensure full reproducibility?

No

Are the conclusions drawn adequately supported by the results?

Yes

Are sufficient details of methods and analysis provided to allow replication by others?

Partly

Reviewer Expertise:

I am an ELSI (Ethical, Legal, and Social Implications of Genetics and Genomics) researcher, with a focus on the lived experiences of individuals undergoing gene therapy and the integration of this therapy into clinical practice.

I confirm that I have read this submission and believe that I have an appropriate level of expertise to confirm that it is of an acceptable scientific standard, however I have significant reservations, as outlined above.

# Response

**Published:** 2026-01-20

**Authors:** Phaedra Locquet

**Affiliations:** KU Leuven Department of Public Health and Primary Care, Leuven, Flanders, Belgium

We thank the reviewer for the careful evaluation of our manuscript. We have addressed each comment in detail, and our responses are presented below.

1. We agree that the use of the term ‘curative’ is debated in the literature and that long-term evidence is still limited. We have therefore adjusted the wording in the manuscript to reflect this nuance, noting that gene therapy is often presented as curative while emphasizing its potentially durable, and briefly acknowledging the ongoing discussion around this framing. *“Although gene therapy is most often presented as a curative treatment, a framing that remains debated given limited long-term follow-up, its effects are currently considered potentially durable, and attitudes towards its preventive use remain unclear.”*
2. We have added additional details related to the provided information to the Methods section, as follows: *“More specifically, the animated video provided information on the nature and modification of genetic and the origin of genetic disorders. It also explained how gene therapy targets the genetic mutation, the use of (viral) vectors and the therapeutic aim of the treatment.”* However, we did not provide extensive details on the mechanisms underlying gene therapy or on more complex aspects of the intervention. While we acknowledge that this information is important, we aimed to keep the informational video as concise as possible due to the recruitment strategy, which limited the opportunity to include more in-depth explanations. Therefore we focussed on the genetic nature of the intervention in a broad sense. We have now also included this in the limitations of the study, as follows: *“Additionally, the informational video was necessarily brief due to the recruitment strategy, focusing primarily on the genetic nature of gene therapies. As a result, detailed information on complex delivery procedures and gene therapy mechanisms was not included, which may have influenced participants’ perceptions.”*
3. We agree that information related to symptoms, side-effects, was missing from the paper. Therefore, we have added, for each table, the specific case that was presented to the participants when answering. These cases clearly described the impact of the symptoms. One example is provided below: *“Results related to the following case: “Suppose you have a genetic disease. The symptoms of your illness strongly affect your daily life. You experience difficulties performing household tasks, have problems with mobility, face limitations in social activities, and you are unable to sustain a full workday. Your condition also worsens with age, increasingly impacting your life. There is now a new gene therapy available which, as the only treatment, can stop the progression of your disease. However, because this therapy is new, scientists still know little about its effectiveness, long-term effects, and possible side effects.”* However, as this paper aimed to gain a broader understanding of participants’ perceptions of gene therapy, we did not include specific side effects in order to limit the number of survey questions and associated survey fatigue. Moreover, we specifically aimed to assess their perceptions of the associated (long-term) uncertainty of these therapies.
4. We followed the case design of Ormondroyd et al. (2024), who used cases involving individuals aged 5, 20, and 50 years. After discussion within the research team, we decided to include an age level of 65 years instead of 50 years to better reflect greater differences in life stage, particularly in terms of responsibilities and social context.
5. We agree that recruiting participants through pharmacies may not fully capture the perspectives of the broader public, as these individuals might have higher health awareness or greater receptivity to medical interventions. We have added a note in the limitations section acknowledging this potential source of selection bias. We have added the following text to the limitations: *“Recruitment through pharmacies and the use of convenience sampling may have introduced selection bias, as participants visiting pharmacies could have higher health awareness or greater receptivity to medical interventions. Additionally, the sample overrepresented Catholic women and predominantly consisted of highly educated individuals, which may have influenced responses related to information and trust in science.”*

# Reviewer report: Reviewer response for version 1

**DOI:** 10.21956/openreseurope.23216.r63843

**Published:** 2025-11-20

**Authors:** Sharon Phares

**Affiliations:** 1 Tufts Medical Center, Boston, Massachusetts, USA

This was a solid paper on a very interesting topic. A few minor changes are warranted:

 1) In the introduction the authors mention low clinical trial participation without the primary reason - that these therapies almost always target very small populations, many of whom have huge time delays in obtaining a diagnosis and are often faced with life-threatening disease. 

 2) Additionally, the statement that 'gene therapy is most often presented as a curative treatment' is more the media presenting them this way. More accurately, they should be presented as durable in effect but there has not been enough time for patient follow-up to claim 'curative' in the lifetime sense.

Is the study design appropriate and does the work have academic merit?

Yes

Is the work clearly and accurately presented and does it cite the current literature?

Partly

If applicable, is the statistical analysis and its interpretation appropriate?

Yes

Are all the source data underlying the results available to ensure full reproducibility?

No

Are the conclusions drawn adequately supported by the results?

Yes

Are sufficient details of methods and analysis provided to allow replication by others?

Yes

Reviewer Expertise:

I have done research and published extensively in the area of gene therapy.

I confirm that I have read this submission and believe that I have an appropriate level of expertise to confirm that it is of an acceptable scientific standard.

# Response

**Published:** 2026-01-20

**Authors:** Phaedra Locquet

**Affiliations:** KU Leuven Department of Public Health and Primary Care, Leuven, Flanders, Belgium

We thank the reviewer for the careful evaluation of our manuscript. We have addressed each comment in detail, and our responses are presented below.

1. We agree that the small size of target populations and delays in diagnosis are primary drivers of low clinical trial participation in gene therapy. We have revised the introduction to explicitly acknowledge these factors before discussing additional ethical and methodological challenges related to trial participation and inclusion criteria. The text now reads as follows: *“Because many gene therapies target very small, rare disease populations, and because these patients often experience significant delays in obtaining an accurate diagnosis while facing life-threatening conditions, clinical trial recruitment is inherently limited. Beyond these structural constraints, low participation in clinical trials remains persistent, as many eligible individuals hesitate due to unknown long-term effects, adverse side effects, and the therapy’s experimental status.”*
2. We agree that it is premature to characterize gene therapies as definitively ‘curative’ in a lifetime sense, given the limited long-term follow-up. We have revised the manuscript to describe these therapies as having potentially durable effects, rather than as curative. *“Although gene therapy is most often presented as a curative treatment, a framing that remains debated given limited long-term follow-up, its effects are currently considered potentially durable, and attitudes towards its preventive use remain unclear.”*
